# Comparison of the Effect of Silver Nanoparticles Biosynthesized with *Lavandula angustifolia* Extract and Lavender Essential Oil Against Multidrug-Resistant and Biofilm-Forming *Staphylococcus* Species

**DOI:** 10.3390/pharmaceutics18080930

**Published:** 2026-07-29

**Authors:** Patrícia Hudecová, Silvia Ondrašovičová, Vanda Hajdučková, Nikola Dančová, Gabriela Gregová, Lívia Mačák, Oksana Velgosová, Jana Ondrašovičová, Ján Király

**Affiliations:** 1 Department of Microbiology and Immunology, The University of Veterinary Medicine and Pharmacy in Košice, 041 81 Košice, Slovakia; patricia.hudecova@student.uvlf.sk (P.H.); vanda.hajduckova@uvlf.sk (V.H.); 2 Department of Biology and Physiology, The University of Veterinary Medicine and Pharmacy in Košice, 041 81 Košice, Slovakia; silvia.ondrasovicova@uvlf.sk; 3 Department of Public Veterinary Medicine and Animal Welfare, The University of Veterinary Medicine and Pharmacy in Košice, 041 81 Košice, Slovakia; nikola.dancova@uvlf.sk (N.D.); gabriela.gregova@uvlf.sk (G.G.); 4 Faculty of Mechanical Engineering, Technical University of Košice, Letná 9, 042 00 Košice, Slovakia; livia.macak@tuke.sk (L.M.); oksana.velgosova@tuke.sk (O.V.); 5 Department of Stomatology and Maxilofacial Surgery, Faculty of Medicine, Pavol Jozef Šafárik University in Košice, 041 90 Košice, Slovakia; jana.ondrasovicova@upjs.sk

**Keywords:** nanoparticles, silver, green synthesis, lavender essential oil, *Lavandula angustifolia*, multidrug resistance, biofilm, *Staphylococcus* spp.

## Abstract

**Background**: Multidrug-resistant and biofilm-forming staphylococci pose a threat to the sustainability of public health, livestock health and the ecosystem. Pathogenic potential with a worsening prognosis of therapy is mainly due to methicillin-resistant *Staphylococcus aureus* (MRSA) or multidrug-resistant Non-*aureus* staphylococci and mammaliicocci (NASM). Alternative approaches based on the use of biosynthesized nanoparticles or substances of natural origin appear to be promising solutions to the problem of ineffective suppression of infections caused by pathogenic microorganisms. **Methods**: The aim of this study was to monitor and compare the biological effects of silver nanoparticles prepared by green synthesis using *Lavandula angustifolia* and lavender essential oil. In particular, the antibacterial, antibiofilm, and biofilm-eradicating effects against biofilm-forming and multidrug-resistant reference strains and field isolates of staphylococci were monitored. **Results**: AgNPs inhibited the growth and formation of biofilms of *S. aureus* strains at a concentration of 0.05 μg/μL, but clinical NASM at 0.025 μg/μL. Sensitivity to Lavender essential oil (LEO) was the same against all tested staphylococcal strains, with an antibacterial MIC of 0.901 μg/μL. The essential oil also had an effect on biofilm formation against all tested strains, but its effect was recorded at a tenfold lower concentration (antibiofilm MIC = 0.0901 μg/μL). No eradication activity was recorded for either tested substance. Their activity against the formed biofilms was not recorded. **Conclusions**: The results demonstrate promising antibacterial and antibiofilm activities of biosynthesized AgNPs and lavender essential oil under in vitro conditions. These findings support further investigation of these materials as potential alternative antimicrobial approaches, particularly in combination with conventional antimicrobial agents.

## 1. Introduction

Microorganisms have the ability to adapt to the immune system of their host and to the toxic effects of drugs. The highest degree of adaptability is shown by resistant bacteria and some rapidly mutating viruses [1,2]. In the background of these changes, a whole series of processes are taking place, providing mechanisms including horizontal gene transfer, biofilm formation and high mutation rates, which are especially common in viruses. All evolutionary strategies ultimately aim at such changes in the organism as to maximize the probability of its survival after the action of stress factors. This is the essence of their escape from host immunity with a significant impact on the clinical course of infection or the effect of vaccines and drugs. Therefore, whenever a resistant variant is detected, there is an effort to understand the nature of this change and, based on the results obtained, to prevent it, to innovate therapeutic approaches and to introduce new strategies in this fight. In the case of viral infections, there is an effort to determine the most effective vaccination strategy, to prepare the most effective antivirals or immunomodulatory systems [3,4,5]. However, due to their biological nature as prokaryotic organisms, research on bacteria is primarily focused on the development of new antimicrobial agents and substances that enhance the effect of existing drugs to which resistant strains have been identified [6].

Antibiotics are still the main way to eliminate bacterial pathogens. Therefore, the rise in new strains of super bacteria characterized by multidrug resistance (MDR) with different mechanisms of action is of great concern. Antibiotics represent a major stress factor for bacteria, inhibiting their growth and viability while also acting as a selective evolutionary pressure. This can result in the stimulation of bacterial mutagenesis and the subsequent emergence of de novo resistance mutations, horizontal transfer of genetic elements or the triggering of phenotypic responses that increase drug tolerance [7]. Bacteria characterized by the ability to evade the effects of antibiotics have been categorized as “ESKAPEE” (*Enterococcus faecium*, *Staphylococcus aureus*, *Klebsiella pneumoniae*, *Acinetobacter baumannii*, *Pseudomonas aeruginosa*, *Enterobacter* spp. and *Escherichia coli*) [8]. Major bacterial pathogens have been classified by the World Health Organization (WHO) into critical, high and medium priority groups to highlight the need for research and development of new antibacterial agents [9].

Bacteria exhibiting antimicrobial resistance (AMR) are associated with the highest burden and healthcare costs not only in humans but also in animals. The most sensitive livestock sector to the negative impact of infectious diseases is cattle farming. Dairy cows play a key role in meeting the growing global demand for food. High financial resources are spent on the treatment of respiratory and musculoskeletal diseases in cattle [10,11]. However, the most significant economic losses and impact on human health are caused by infectious diseases of the mammary gland. Bovine mastitis is a multifactorial disease resulting from inflammation of the mammary gland tissue, manifested by physical, chemical and bacteriological changes. Infectious causes are the most common reason for the development of mastitis and, according to published studies, bacterial pathogens play a primary role in its spread in the herd [12]. No single bacterial genus can be attributed to a unique and exclusive contribution to its development. However, based on published works, it can be stated that some staphylococcal species have a dominant position as key etiological agents. It is currently known that within staphylococci, in addition to the well-known pathogenic species *S. aureus*, species referred to as NASM (Non-*aureus* staphylococci and mammaliicocci) are also significant causes of mastitis [13,14]. Unlike standard laboratory strains, NASM isolates represent clinically relevant mastitis-associated pathogens frequently associated with bovine intramammary infections. Their multidrug resistance and strong biofilm-forming ability make them particularly suitable for evaluating the antimicrobial potential of alternative therapeutic approaches under conditions that more closely reflect veterinary clinical practice. NASM are of great interest due to their frequent occurrence in investigations of intramammary infections. They have been found in the udder in the teat canal area and on the tips of the teats, but also in tank milk, bedding, droppings and have even been isolated from aseptically collected milk samples from quarters [15]. The reason for their relatively wide occurrence is due to the properties they possess. They have the ability to adhere to surfaces and form biofilms, and are capable of ‘hiding’ from the immune system. In addition, they exhibit AMR and contain several resistance genes that may be shared with other species [16,17,18], suggesting that they serve as a reservoir of not only resistance genes but also virulence genes [19]. In connection with these findings, it is important to emphasize that NASMs exhibiting MDR have already been identified in several scientific papers [20]. MDR is a result of the massive use of antibiotics in treatment, and NASM is also assumed to occur on body surfaces, making them more exposed to antimicrobial agents [21].

The solution to the AMR problem may be a more efficient and rational use of effective antimicrobial agents and the search for alternative approaches or their mutual combination using the synergistic effect to enhance the effect. An approach using nanotechnology through nanoparticles (NPs) has proven to be a suitable strategy. Bioactive substances of natural origin, which represent secondary plant metabolites containing terpenes, phenols, alkaloids and polysaccharides contained in essential oils, also have great support in the development of therapeutics [22].

Within the use of NPs, the most studied antibacterial effects of metal NPs are those of silver (Ag), copper (Cu), selenium (Se), nickel (Ni) and gold (Au). They can act against resistant pathogens that have developed mechanisms of target site modification, promoting drug efflux through membranes and increasing the expression of efflux pumps, along with enzyme inactivation and reducing membrane permeability [23]. Research results show that NPs with sizes ranging from 10 to 100 nanometers exhibit unique chemical, physical, and biological properties that distinguish them from other materials. NPs can be classified into different categories depending on their physicochemical properties, such as size, shape, and surface charge. Silver nanoparticles (AgNPs) have the greatest application prospects. Silver has long been used as an antibacterial agent against many bacterial species, and one of its main advantages is its low cytotoxicity [24]. The antibacterial mechanisms of AgNPs are well understood, and their essence is that upon contact with bacteria, the NPs localize to the bacterial cell wall and oxidize, which leads to concentrated release of Ag at the interface between the NPs and the cell [25], which destroys it due to the formation of free radicals. In addition, AgNPs can form silver ions, which, after binding to cellular organelles, cause their dysfunction [26]. Nanoparticles can be synthesized by physical, chemical or biological methods. Traditionally used physical and chemical methods are problematic in terms of cost, time and environmental impact. Therefore, biological methods, referred to as green synthesis, have been used in synthesis. Non-toxic and environmentally friendly reagents are used in the production of AgNPs. Extracts derived from organisms that are rich in biomolecules are used [27].

An alternative therapeutic approach represents the use of essential oils (EOs) extracted from aromatic plants. Oils are volatile and fragrant concentrated plant extracts obtained from various parts of the plant, including leaves, roots, flowers, fruits, resin, seeds and bark. They contain a complex hydrophobic mixture of hundreds of organic compounds that exhibit potent antioxidant, antiparasitic, antiviral and antibacterial effects [22,28]. The potential for their use lies in the combination of EO with antibacterial agents. They can thus enhance their inhibitory activity or, through the synergistic effect of the active substances, allow for a dose reduction with a direct impact on reducing side effects. Lavender essential oil (LEO) extracted from the flowering tops of *Lavandula angustifolia* Mill. (*Lamiaceae*) is a promising candidate for a natural product, for which pharmacological effects including antibacterial, antifungal, antioxidant, anxiolytic, but also anticonvulsant and anticholinesterase properties have been described [29]. Its antibacterial and antifungal effects can be attributed to the high content of its main components, which include linalool, linalyl acetate, lavandulol, geraniol or eucalyptol [30].

This study investigates the effect of AgNPs biosynthesized with *Lavandula angustifolia* extract and compares them with the effect of LEO against biofilm-forming and multidrug-resistant reference strains and field isolates of staphylococci isolated from sick animals. The aim was to determine the potential and differences in activity of individual alternative approaches to standard antibiotic treatment based on efficacy results. These findings are an important prerequisite for the next part of the planned research focused on synergistic approaches in combination therapeutic procedures.

## 2. Materials and Methods

### 2.1. Selection of Bacterial Strains for Testing

To evaluate the antibacterial, antibiofilm and biofilm eradication effects of AgNPs and LEO, two reference strains of *S. aureus* obtained from the Czech Collection of Microorganisms (Brno, Czech Republic) were selected. These were *S. aureus* CCM 4750, which is characterized as methicillin-resistant, and *S. aureus* CCM 4223, which represents a strong biofilm producer. Other tested strains belong to the collection of the Department of Microbiology and Immunology of the UVLF in Košice (Slovakia) and represent clinical isolates of Non-*aureus* staphylococci and mammaliicocci (NASM), which were identified as multiresistant and at the same time as strong biofilm producers. The important causative agent of bovine mastitis, *Mammaliicoccus sciuri* (originally *S. sciuri*), and the opportunistic pathogen causing skin infections, *S. pseudintermedius,* were selected. These bacterial strains were deposited in Genbank and received the accession numbers of PX904985 (*S. sciuri*) and PV688034 (*S. pseudintermedius*). Their properties were described in detail in Király et al. [31].

### 2.2. Materials

Silver nitrate (AgNO_3_ > 98% purity) supplied by Mikrochem Ltd. (Pezinok, Slovakia) was used as the silver precursor for NP synthesis. Dried leaves of *Lavandula angustifolia* (lavender) were used as the plant material for extract preparation. All experimental solutions were prepared using deionized water.

### 2.3. Preparation of Lavender Extract

Dried lavender leaves were used for extract preparation. Prior to extraction, the plant material was washed twice with distilled water to remove impurities and dust particles. Subsequently, the leaves were mechanically crushed using a mortar in order to disrupt the cell walls and facilitate the release of biologically active compounds.

Approximately 2.5 g of dried lavender leaves were mixed with 100 mL of deionized water. The mixture was heated at 70–80 °C for 10 min under constant stirring at 600 rpm. After cooling, the extract was filtered through filter paper to remove solid residues and centrifuged at 9000 rpm for 10 min to obtain a purified extract. The prepared extract was stored at 4 °C and used for AgNP synthesis within 5 days.

The prepared lavender extract was subjected to additional physicochemical charac-terization using Fourier-transform infrared spectroscopy (FTIR) (Bruker Tensor 27 spectrometer, Bruker Optics, Ettlingen, Germany) and high-performance liquid chromatography coupled with diode-array detection (HPLC-DAD) (Agilent 1260 Infinity Quaternary LC system equipped with a 1260 Infinity DAD detector, Agilent Technologies, Santa Clara, CA, USA) in order to identify bioactive compounds and functional groups potentially responsible for the reduction and stabilization of silver nanoparticles. A detailed description of the analytical procedures and the corresponding results is presented in the Supplementary Materials (Figures S1 and S2).

### 2.4. Preparation of AgNP Colloids

Silver nanoparticle colloids were prepared using a green synthesis approach. A precursor solution of Ag^+^ ions with a concentration of 50 mg/L was prepared from AgNO_3_. The precursor solution was heated to 70–80 °C under constant stirring at 600 rpm. After reaching the desired temperature, the lavender extract was added to the precursor solution in a precursor-to-extract volume ratio of 10:2.

Following extract addition, the reaction mixture was maintained at approximately 70 °C for an additional 20 min under continuous stirring. During the synthesis process, a visible color change in the solution from light brown to dark brown was observed, indicating the reduction of Ag^+^ ions and the formation of AgNPs.

### 2.5. Characterization Methods

The formation and stability of AgNPs were monitored using UV–Vis spectrophotometry (UNICAM UV4, Waltham, MA, USA) in the wavelength range of 350–700 nm. Measurements were performed at room temperature using 1 cm quartz cuvettes. The appearance of the characteristic surface plasmon resonance (SPR) absorption band confirmed the formation of AgNPs. The morphology and particle size distribution of the synthesized NPs were investigated using transmission electron microscopy (TEM, JEOL JEM-2000FX, Tokyo, Japan) operated at 200 kV. Surface morphology was additionally analyzed using scanning electron microscopy (SEM) (SEM/FIB ZEISS-AURIGA Compact, Jena, Germany).

### 2.6. Lavender Essential Oil (LEO)

Lavender essential oil (extracted from *Lavandula angustifolia*) was obtained from Calendula a.s. (Nová Ľubovňa, Slovakia). The main components were determined by the manufacturer by gas chromatography/mass spectrometry (GC/MS), and their chemical composition is listed in Table 1.

### 2.7. Preparation of Bacterial Strains for Testing

Bacterial strains for AgNPs and LEO testing were cultured overnight for 18 h at 37 °C on blood agar (HiMedia, Mumbai, India). After incubation, individual colonies were resuspended in physiological solution to achieve a turbidity standard of 1 McFarland.

### 2.8. Antibacterial Activity of AgNPs and LEO

The antibacterial activities of AgNPs and LEO were evaluated by determining the minimum inhibitory concentration (MIC). Testing followed the guidelines of the European Committee on Antimicrobial Susceptibility Testing (EUCAST 2025, version 15.0) [32] and the Clinical Laboratory Standards Institute (CLSI, M100-S16) [33]. The antibacterial activity of the tested AgNPs and LEOs was evaluated after their dilution in modified BHI (mBHI—Brain Heart Infusion Broth, HiMedia Laboratories, Mumbai, India) containing 1% glucose and 2% NaCl. Subsequently, the respective dilutions were incubated with bacterial strains in a 96-well microtiter plate (Brand, Wertheim, Germany). Various AgNP concentrations from 25 to 200 μg/mL and LEO concentration ranges from 0.001 to 1% were tested. Since EOs are lipophilic, they were processed to obtain a homogeneous mixture with the water-based bacterial suspension using Tween 20 (AppliChem, Darmstadt, Germany).

Individual concentrations of AgNPs and LEO were prepared to reach the desired final concentration after two-fold dilution with bacterial culture, as the well volume was 200 μL and contained 100 μL of the respective AgNPs or LEO dilution and 100 μL of bacterial inoculum. Each dilution was tested in triplicate. The final bacterial density in wells containing either AgNPs or LEO was 0.5 McFarland. Control wells used for background subtraction contained broth with AgNPs or LEO along with physiological solution. Pure broth and physiological solution with bacterial strains served as positive controls for bacterial growth. The negative control was pure broth with physiological solution (without bacterial culture). After 24 h of incubation at 37 °C with constant shaking, growth inhibition was measured spectrophotometrically by recording the absorbance at 600 nm using a Synergy reader 4 (Merck, Darmstadt, Germany). MIC value was defined as the lowest dilution at which no visible growth of the microorganism occurred (i.e., no turbidity).

### 2.9. Antibiofilm Activity of AgNPs and LEO

Biofilm inhibition by NPs or LEO was tested in microtiter plates with 100 μL of bacterial suspension (1 McFarland) and 100 μL of mBHI containing selected concentrations of NPs or EO. These concentrations matched those in the bacterial growth inhibition assay and were evaluated in triplicate. A bacterial suspension without NPs or EO served as a positive control for biofilm formation. Broth alone was used as a negative control, and pure NPs or EO at appropriate dilutions without bacteria were used for background reading. Plates were incubated at 37 °C for 24 h. Biofilm activity was then assessed using a modified O’Toole protocol [34]. Inhibition was measured spectrophotometrically at 550 nm using a Synergy reader 4 (Merck, Darmstadt, Germany).

### 2.10. Effect of AgNPs and LEO on Biofilm Eradication

The effect of AgNPs and LEO on biofilm eradication was monitored at the same selected concentrations as in previous experiments in this study. Bacterial suspensions (1 McFarland) and mBHI were mixed in microtiter plate wells at a ratio of 1:1 and incubated for 24 h at 37 °C. After the formation of a continuous biofilm, the mBHI was replaced with fresh mBHI medium containing the selected concentrations of the test substances. The plates were incubated again at 37 °C for 24 h. After incubation, the biofilm was evaluated using a modified O’Toole protocol [34]. The presence of biofilm was determined by measuring the absorbance at 550 nm using a Synergy reader 4 (Merck, Darmstadt, Germany).

### 2.11. Statistical Analysis

The assessment of antibacterial, antibiofilm and biofilm eradication activity was performed using statistical analysis by performing one-way ANOVA with Dunnett’s test using Prism 8.3.0 software (GraphPad Software, San Diego, CA, USA). All tests were performed in triplicate, and the mean ± SD was calculated for each isolate.

## 3. Results

In this work, the antibacterial, antibiofilm and biofilm-eradicating effects of biosynthesized NPs via *Lavandula angustifolia* extract and LEO extracted from *Lavandula angustifolia* were evaluated and compared to determine their potential use as an alternative approach for the treatment of bacterial infections.

### 3.1. AgNPs Biosynthesis and Characterization

Silver nanoparticles were synthesized via a green approach using an aqueous extract of *Lavandula angustifolia* leaves as a reducing and stabilizing agent. The plant extract, rich in polyphenols and other bioactive compounds, enabled efficient reduction of Ag^+^ ions under mild conditions (80 °C, 20 min) without the use of additional chemical reagents. The formation of AgNPs was confirmed by UV–Vis spectroscopy, showing a characteristic SPR band at ~430–440 nm.

Morphological analysis by TEM revealed predominantly spherical nanoparticles with an average diameter of approximately 15 nm. SEM confirmed the nanoscale morphology, while EDX verified the presence of elemental silver as the dominant component.

Dynamic light scattering (DLS) analysis revealed a hydrodynamic particle diameter of 76.89 nm, while the synthesized AgNPs exhibited a zeta potential of −15.90 mV and a polydispersity index (PDI) of 0.331. These values indicate a moderately polydisperse colloidal system suitable for the performed biological experiments. Detailed DLS size distribution, zeta potential distribution and long-term stability data are provided in the Supplementary Materials (Figure S1).

The obtained results demonstrate that lavender-mediated synthesis provides a simple, cost-effective and environmentally friendly route for the preparation of sufficiently stable AgNPs with potential applications in pharmaceutical and antimicrobial systems.

#### 3.1.1. Mechanism of Green Synthesis

The synthesis of AgNPs using lavender extract is driven by phytochemicals such as polyphenols, flavonoids and terpenoids, which act as both reducing and stabilizing agents [35,36,37]. These compounds donate electrons to Ag^+^ ions, leading to their reduction into metallic Ag^0^ nuclei. Simultaneously, functional groups (–OH, –COOH) present in these biomolecules adsorb onto the NP surface, forming a stabilizing organic layer that prevents uncontrolled aggregation. This dual role explains the formation of relatively uniform and stable NPs without the need for additional surfactants, as reported in other green synthesis studies [38,39].

#### 3.1.2. Optical Properties (UV–Vis)

**Figure 1:**
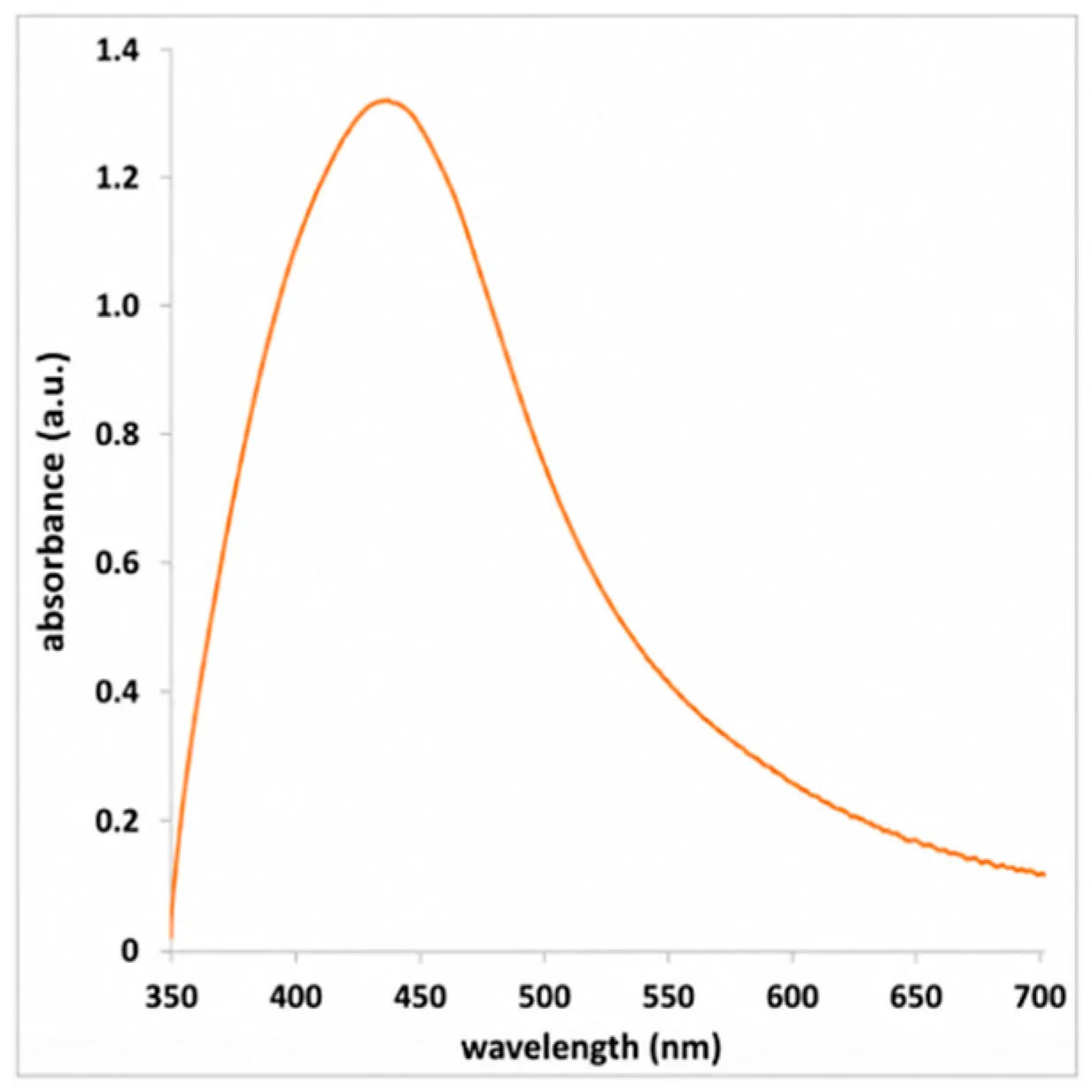
UV–Vis absorption spectrum of AgNPs synthesized using lavender leaf extract, showing a characteristic SPR band at ~430–440 nm.

The UV–Vis spectrum in Figure 1 shows a distinct SPR band at approximately 430–440 nm, which is characteristic of spherical AgNPs [40,41]. The position of the SPR peak indicates particle sizes in the range of tens of nanometres, consistent with TEM observations. The absence of significant peak broadening or red-shift suggests limited aggregation and a relatively narrow size distribution. The gradual tailing toward higher wavelengths may indicate minor polydispersity or weak interparticle interactions.

#### 3.1.3. Morphology and Size Distribution (TEM)

**Figure 2:**
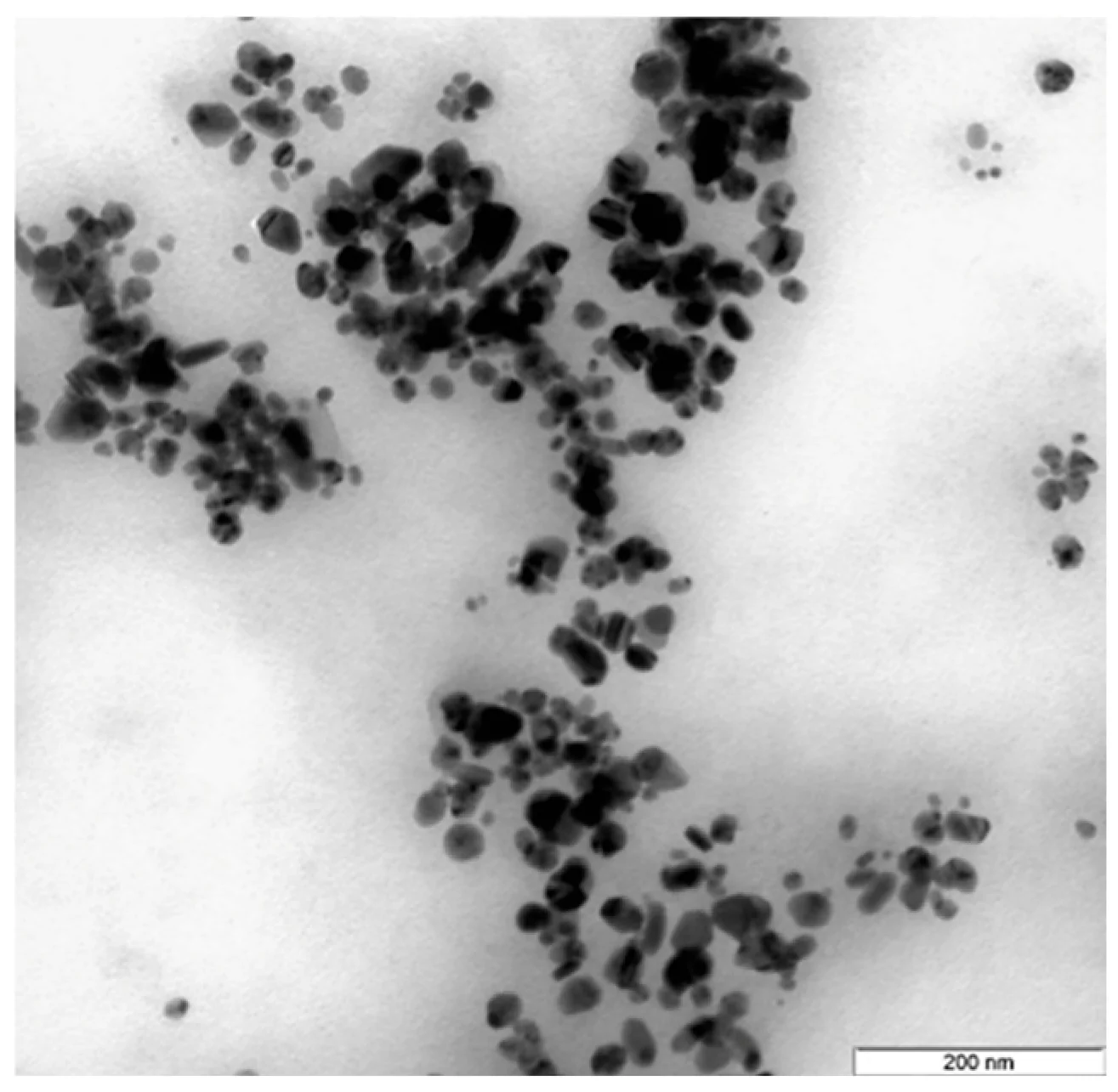
TEM micrograph of AgNPs synthesized via green method using lavender extract, revealing predominantly spherical NPs.

TEM analysis confirms the formation of quasi-spherical AgNPs with good dispersion. The evaluated particle size distribution indicates an average diameter of ~15 nm, with most particles below 25 nm (Figure 2). Comparable NP sizes have been reported for green-synthesized systems, including plant-mediated and alternative biological approaches [42,43].

#### 3.1.4. Surface Morphology (SEM)

**Figure 3:**
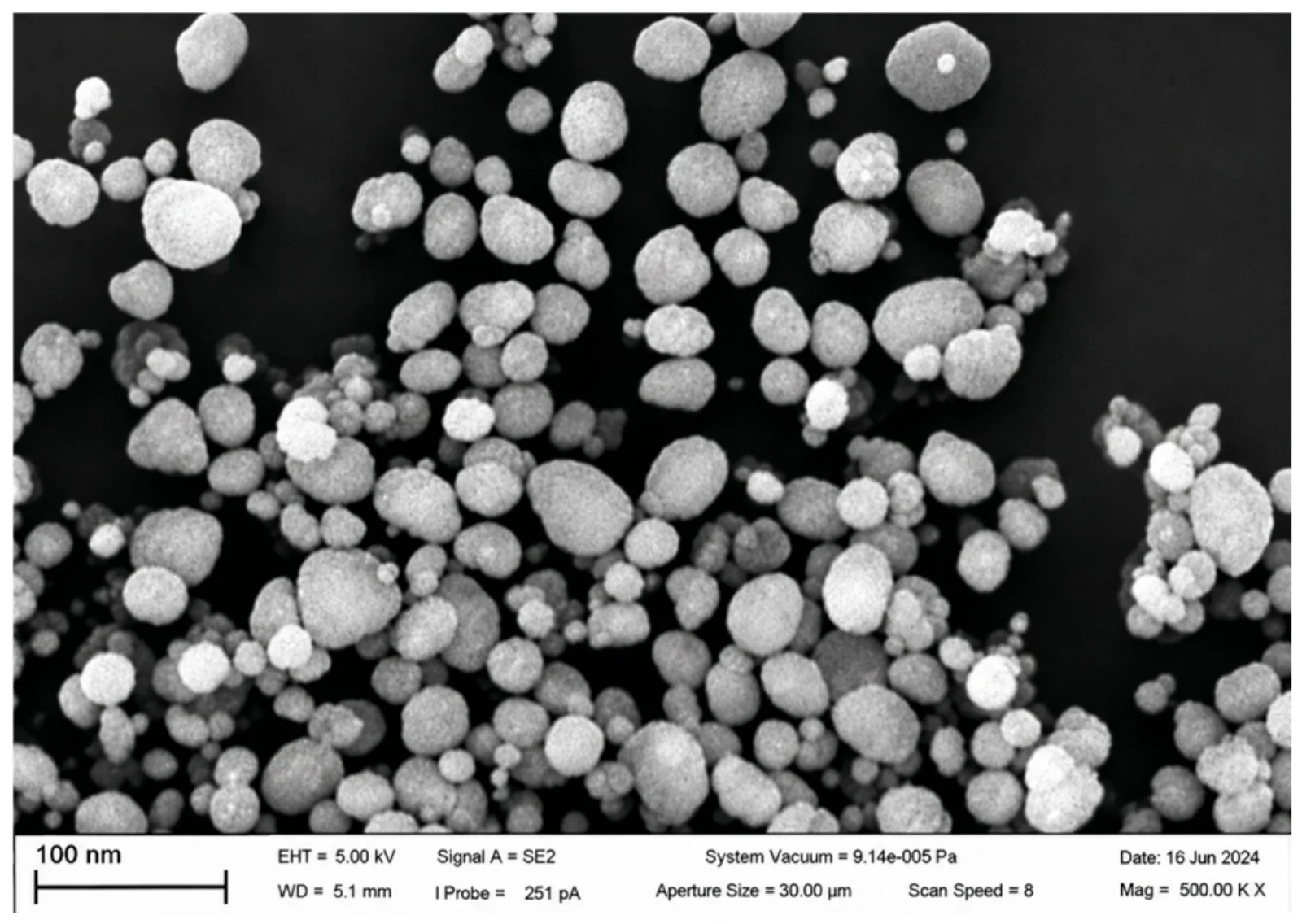
SEM image showing the surface morphology and clustering behavior of AgNPs.

SEM images reveal clusters of nanosized particles with relatively homogeneous morphology. Observed agglomeration is attributed primarily to sample preparation (drying effects), rather than intrinsic instability of the colloidal system (Figure 3). The agreement between SEM and TEM results supports the reliability of the morphological characterization.

#### 3.1.5. Elemental Composition (EDX)

**Figure 4:**
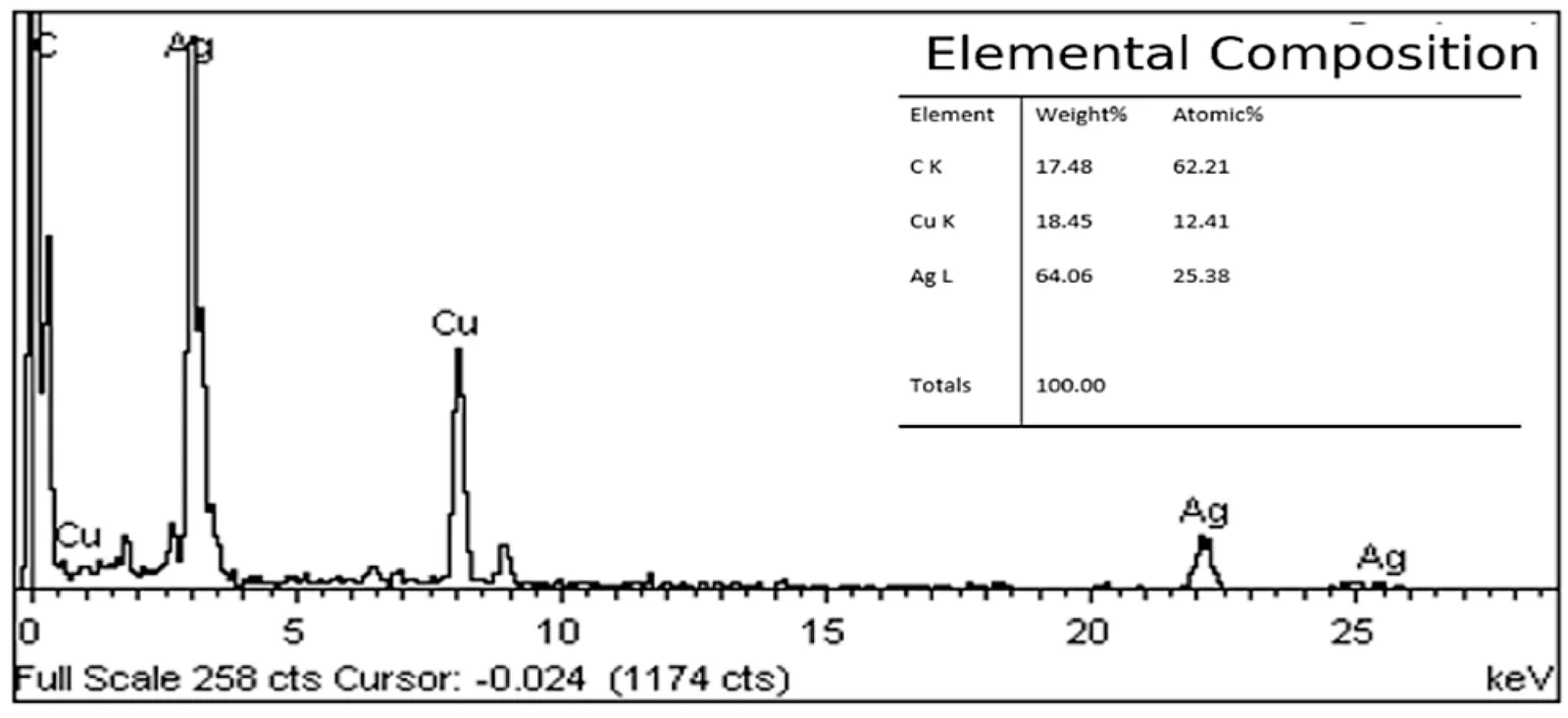
EDX spectrum confirming the presence of elemental silver as the dominant component in the synthesized NPs.

Energy-dispersive X-ray spectroscopy (EDX) analysis confirms the successful formation of AgNPs, with a dominant Ag signal (~64 wt. %). Minor contributions from carbon originate from organic capping agents derived from the plant extract, while copper signals are associated with the sample holder (Figure 4). Similar elemental profiles have been reported in related studies [40,42].

The hydrodynamic size, zeta potential, and polydispersity index (PDI) of the synthesized AgNPs were evaluated to assess their colloidal stability. The obtained values indicate a moderately polydisperse colloidal system that remained sufficiently stable under the storage conditions used in this study. Detailed DLS size distribution, zeta potential profiles, and PDI data are provided in the Supplementary Materials (Figures S1 and S2, and Table S1).

The particle size distribution determined by DLS is consistent with TEM analysis, which confirmed smaller core particle sizes due to the absence of the hydration layer. Furthermore, the long-term colloidal stability was verified by UV–Vis spectroscopy, showing no significant shift or broadening of the localized surface plasmon resonance (LSPR) peak over a period of 21 days. The corresponding spectra are included in the Supplementary Materials (Figure S5).

### 3.2. Evaluation of Antibacterial Activity of AgNPs and LEO

**Figure 5:**
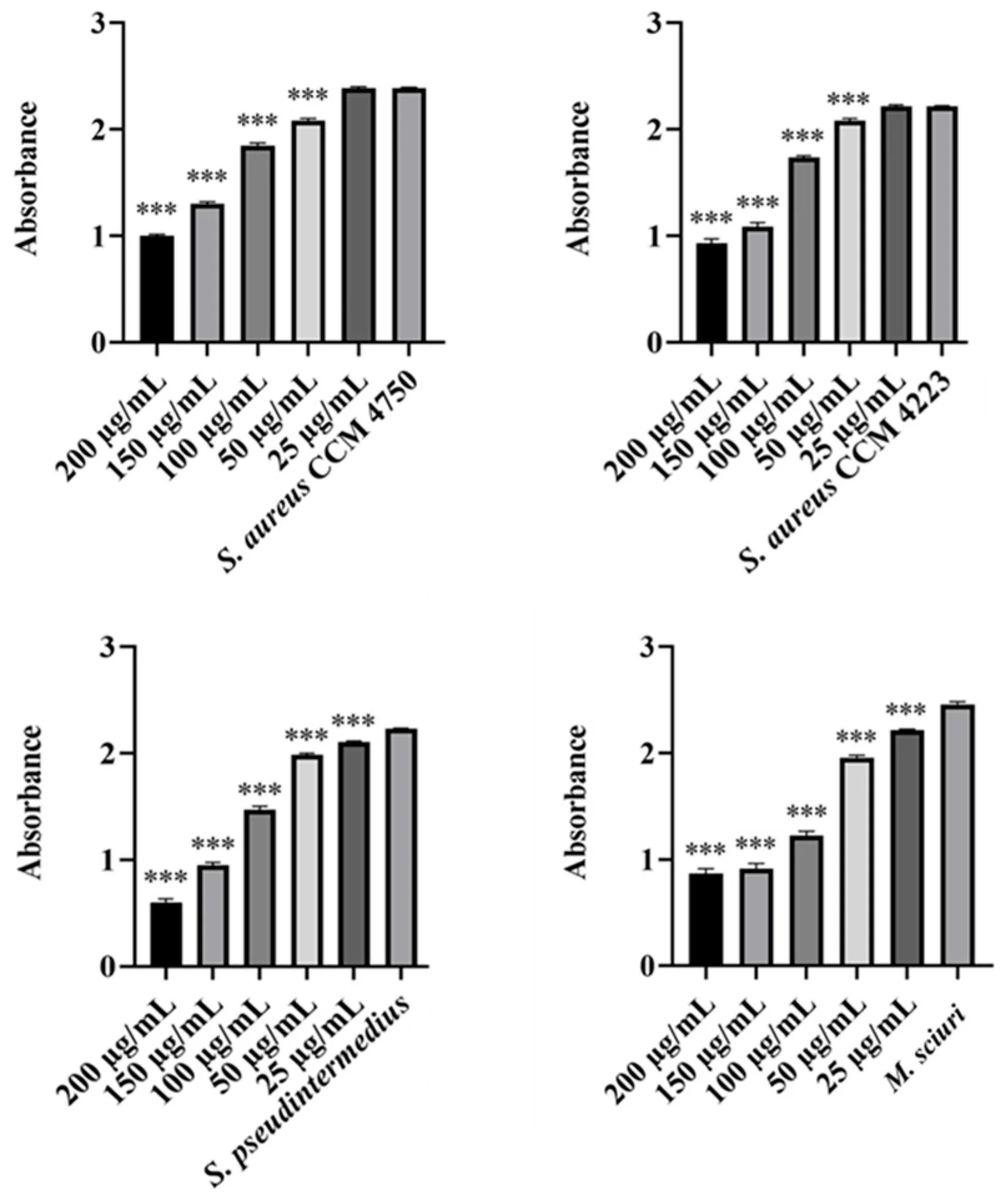
Antibacterial activity of AgNPs against staphylococci. Values are presented as mean ± SD (*n* = 3). Statistical significance was evaluated using one-way ANOVA followed by Dunnett’s test. Detailed statistical results are provided in Supplementary Table S1. *** *p* < 0.001.

The antibacterial activity of AgNPs against the tested strains of staphylococci was not identical. The growth inhibition of both *S. aureus* species was monitored at a concentration of 50 μg/mL, which was calculated as a concentration of 0.05 μg per 1 μL, so the effective concentration against NASM was twice as low (25 μg/mL, or 0.025 μg/μL) (Figure 5). Statistical analysis confirmed significant differences between treated groups with AgNPs and the positive control (Supplementary Table S1).

**Figure 6:**
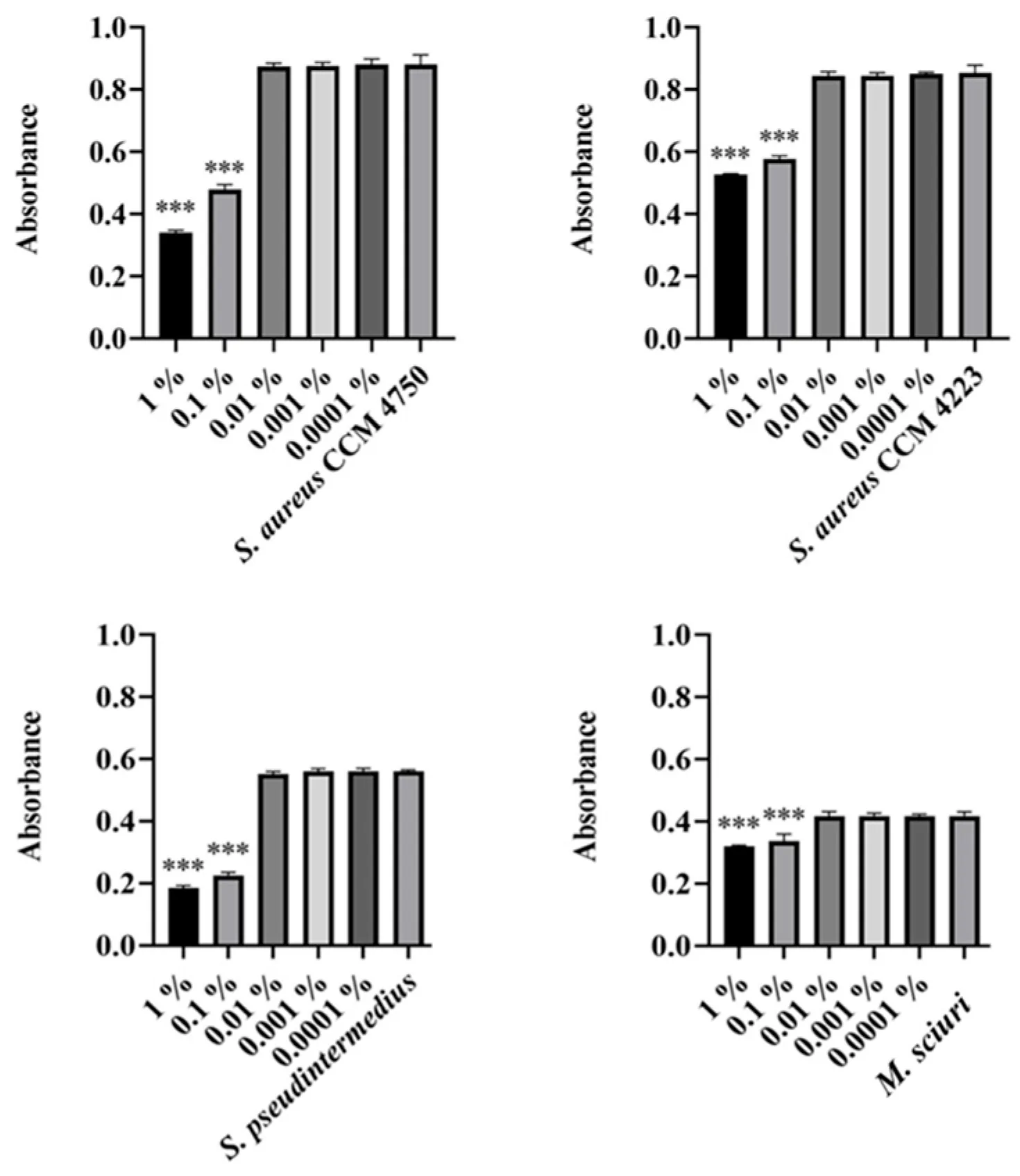
Antibacterial activity of LEO against staphylococci. Values are presented as mean ± SD (*n* = 3). Statistical significance was evaluated using one-way ANOVA followed by Dunnett’s test. Detailed statistical results are provided in Supplementary Table S4. *** *p* < 0.001.

Susceptibility to LEO was demonstrated against all tested staphylococcal strains using the same concentration. The MIC of lavender oil was determined at a dilution of 0.1%, which corresponded to a concentration of 0.901 μg/μL (Figure 6). Statistical analysis is provided in Supplementary Table S4.

### 3.3. Evaluation of Antibiofilm Activity of AgNPs and LEO

**Figure 7:**
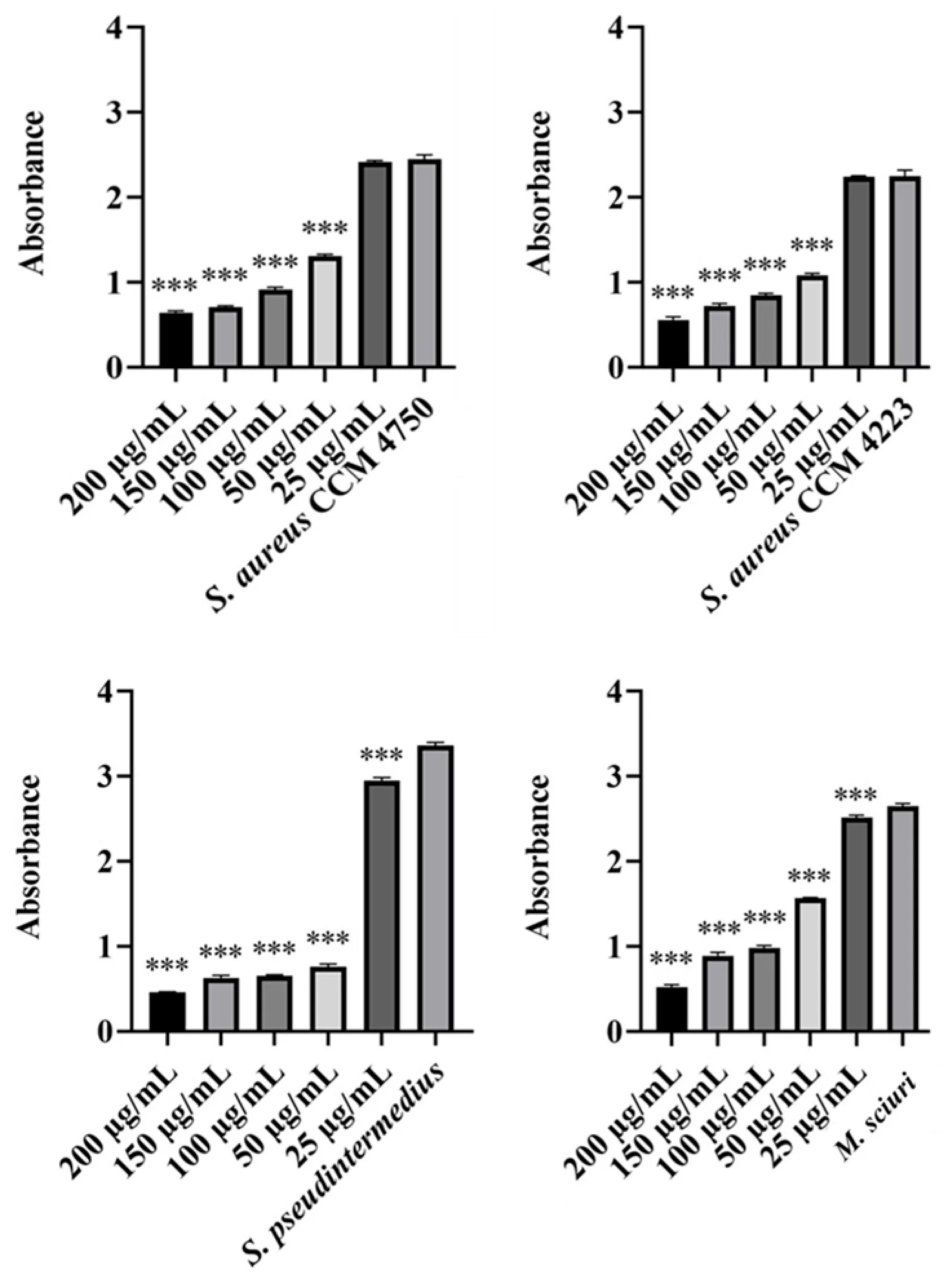
Antibiofilm activity of AgNPs against staphylococci. Values are presented as mean ± SD (*n* = 3). Statistical significance was evaluated using one-way ANOVA followed by Dunnett’s test. Detailed statistical results are provided in Supplementary Table S2. *** *p* < 0.001.

Monitoring the effect of AgNPs on biofilm formation, their inhibitory activity was demonstrated using identical concentrations as for bacterial growth. Biofilm formation in both *S. aureus* strains was significantly suppressed at a concentration of 50 μg/mL (0.05 μg/μL); however, a lower concentration of 25 μg/mL (0.025 μg/μL) was again effective in suppressing biofilm formation in NASM strains (Figure 7). Statistical analysis confirmed significant differences compared to the positive control (Supplementary Table S2).

**Figure 8:**
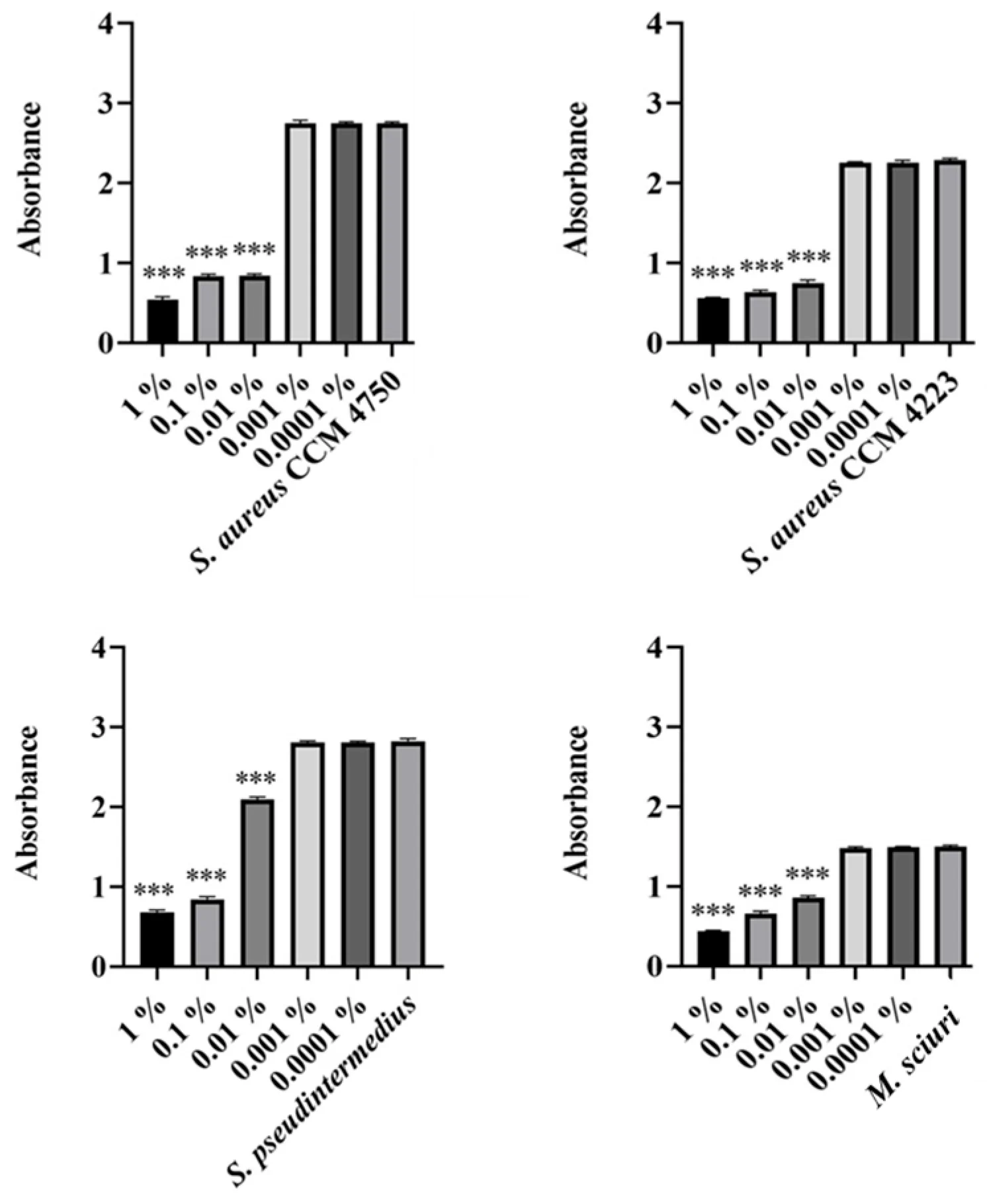
Antibiofilm activity of LEO against staphylococci. Values are presented as mean ± SD (*n* = 3). Statistical significance was evaluated using one-way ANOVA followed by Dunnett’s test. Detailed statistical results are provided in Supplementary Table S5. *** *p* < 0.001.

An interesting result was the observation of a stronger antibiofilm activity of LEO compared to its antibacterial effect. Inhibition of biofilm formation was recorded in all studied strains using a 0.01% concentration of EO, which in terms of mass unit represented 0.0901 μg/μL (Figure 8). Statistical analysis is provided in Supplementary Table S5.

### 3.4. Biofilm-Eradicating Effect of AgNPs and LEO

**Figure 9:**
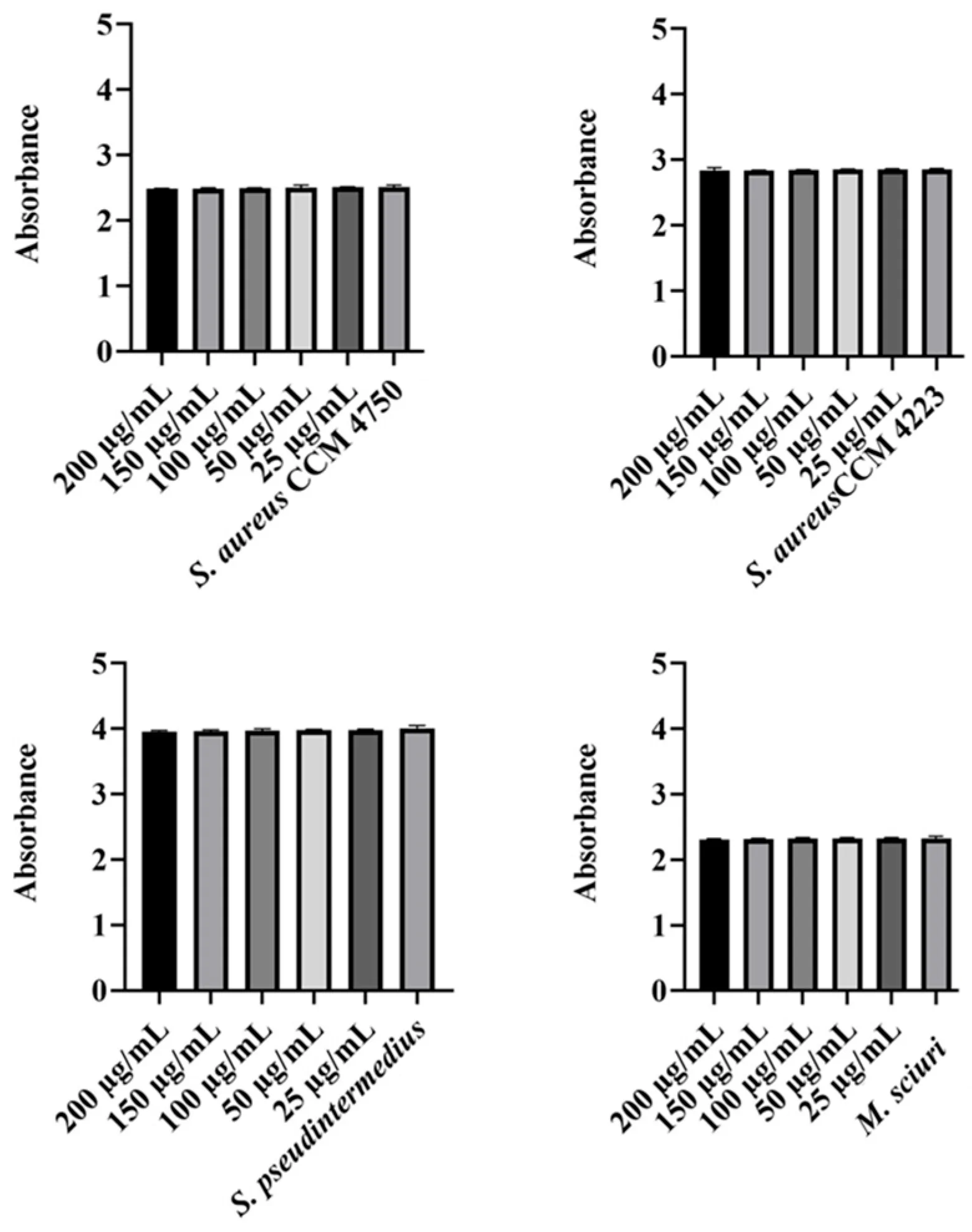
Effect of AgNPs on biofilm eradication against staphylococci. Values are presented as mean ± SD (*n* = 3). Statistical analysis was performed using one-way ANOVA followed by Dunnett’s test. Detailed statistical results are provided in Supplementary Table S3.

**Figure 10:**
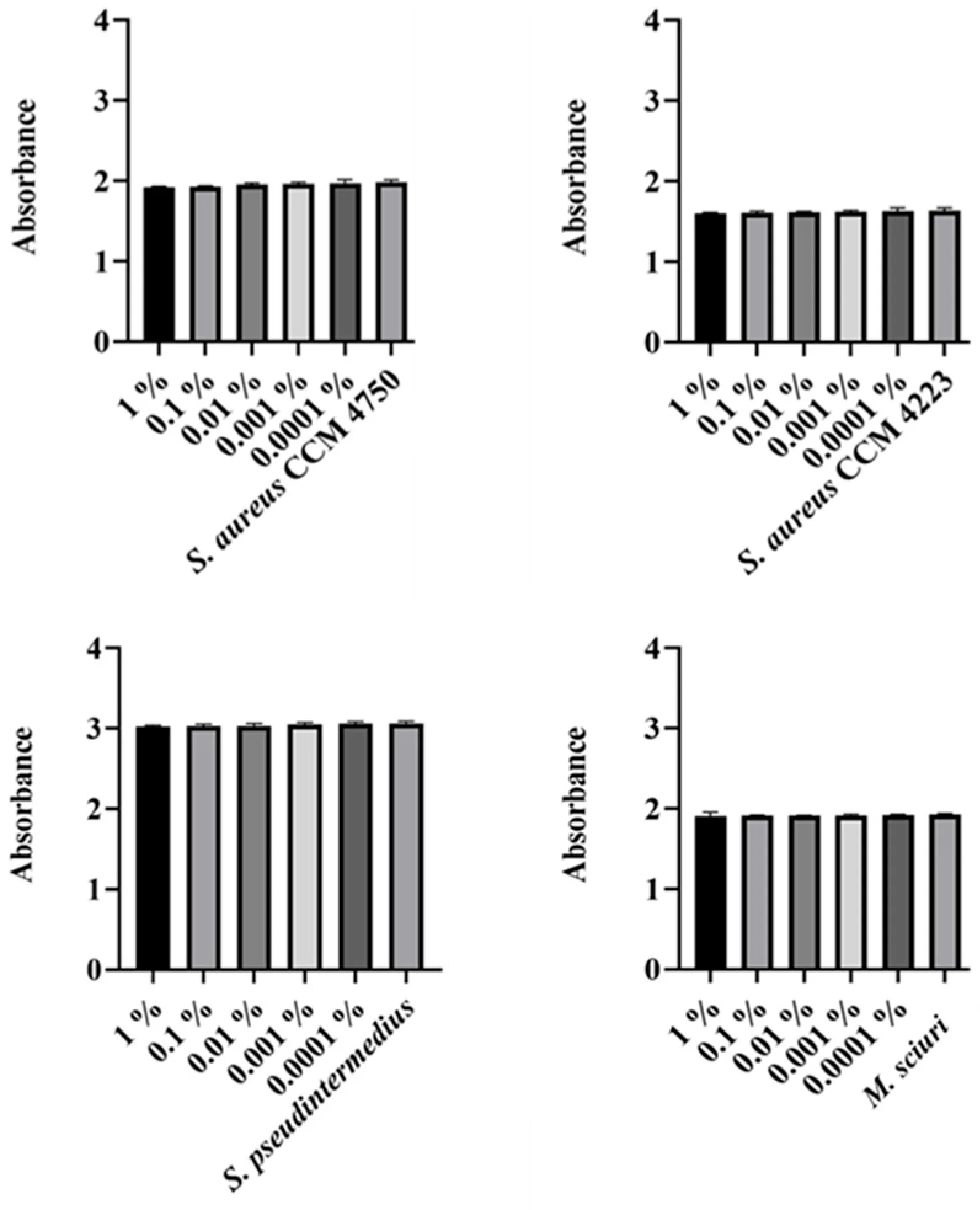
Effect of LEO on biofilm eradication against staphylococci. Values are presented as mean ± SD (*n* = 3). Statistical analysis was performed using one-way ANOVA followed by Dunnett’s test. Detailed statistical results are provided in Supplementary Table S6.

The final goal of the experiments was to monitor the ability of AgNPs and LEO to disrupt the formed biofilm in individual strains of staphylococci. The measurements of the eradication activity of both tested substances did not demonstrate the ability to reduce the amount of formed biofilm. Despite positive results in testing for antibacterial and antibiofilm activity, no effect leading to biofilm eradication was observed, even at high concentrations (Figure 9 and Figure 10). Statistical analysis confirmed the absence of significant differences compared to the positive control (Supplementary Tables S3 and S6).

Based on our obtained results, we evaluated the biological activity of individual substances against significant resistant and biofilm-forming *Staphylococcus* spp. For a better comparison of their efficacy in terms of effective concentrations, the overall results are shown in Table 2.

The lavender extract used for AgNP synthesis did not exhibit any inhibition zones under the applied experimental conditions.

## 4. Discussion

The good adaptive potential of bacteria to the negative impact of stress factors such as the effect of antimicrobial agents leads to the emergence and spread of MDR strains. The WHO states in its report that AMR is a major global challenge for public health and the fight against it requires global efforts focused on several key areas. Significant emphasis is placed on the need to increase investment in research and development aimed at creating new drugs, diagnostics and preventive tools [9]. The most important step in solving this problem is to understand the cause of resistance and its molecular basis. Most antibiotic resistance is the result of lateral transfer of resistance genes between ecologically and taxonomically distinct bacteria [44]. An important property of bacteria from the point of view of colonization and their defense mechanisms is the ability to form a biofilm. Its consistency and complex structure composed of a large number of bacterial cells represent a difficult barrier to overcome for the host immune system and antimicrobial molecules. The result of these adaptations and acquired properties of bacteria is a partial or complete loss of their sensitivity to conventional therapeutic procedures. Alternative approaches are currently largely focused on the use of bioactive substances of natural origin. A significantly positive antibacterial effect was observed with the use of metal NPs, primarily based on silver (AgNPs) and EOs obtained from some plant species.

Numerous studies have investigated and confirmed the biosynthesis of NPs as an effective antibacterial and antibiofilm agent [45,46,47,48]. Thanks to their proven antibacterial properties, AgNPs are already being used in practice for the treatment of surgical materials, filters, or materials used in the food industry. The introduction of the biological synthesis method has not only reduced the use of hazardous chemicals in their production, but also made AgNPs available for biomedical applications [49]. A wide range of plant species have been used in the production of AgNPs, demonstrating the diversity of plant-based approaches. Specific plant metabolites vary significantly depending on the species, which affects the efficiency and characteristics of NP formation [50]. AgNPs synthesized using *Berberis vulgaris* fruit extract showed high antibacterial activity against MDR staphylococci [51]. Strong inhibitory activity against Gram-positive *S. aureus* was also reported by Aisida et al. [52] using AgNPs synthesized with *Vernonia amygdalina* leaf extract in the concentration of 80 μg/mL. Our results in this work show that the lower concentrations used against staphylococci, with significant inhibition of *S. aureus* strains at 50 μg/mL (0.05 μg/μL), and NASM were inhibited at a two-fold lower concentration of 25 μg/mL (0.025 μg/μL).For biosynthesis, we used lavender extract, which we chose because of its easy cultivation and availability. When choosing it, the fact that it has been used since ancient times as an anti-inflammatory and sedative agent was also taken into account. We obtained similar results in an earlier study published by us, but where we monitored the antibacterial effect of AgNPs biosynthesized with an extract from the medicinal plant *Alchemilla vulgaris* [31], with the difference that the MIC for NASM was at a higher concentration (50 μg/mL). When monitoring the antibiofilm activity in this work, we observed a significant inhibition of biofilm formation at identical concentrations as when monitoring bacterial growth and the same result was also observed within the tested strains. Biofilm formation of *S. aureus* strains was suppressed using 50 μg/mL (0.05 μg/μL) and NASM 25 μg/mL (0.025 μg/μL) concentrations. These results were completely consistent with our results obtained using AgNPs biosynthesized with *Alchemilla vulgaris*, where we observed the antibiofilm activity against NASM also at a concentration of 25 μg/mL. A strong antibiofilm effect was also reported by Hamid et al. [53], who used AgNPs synthesized with *Carthamus tinctorius* extract against MDR strains. Hosnedlova et al. [54] reported similar results in the suppression of biofilm formed by staphylococci as we did. In their study, they used AgNPs synthesized from *Lagerstroemia indica* extract and the biofilm was inhibited using 20 μg/mL. In the study by Hosnedlova et al., they also monitored the eradication effect on the formed *S. aureus* biofilm and recorded a 60% reduction when using 20 μg/mL. In our older study, we also monitored the eradication effect of AgNPs synthesized by *Alchemilla vulgaris,* and we recorded significant biofilm degradation using a concentration of 50 μg/mL for both *S. aureus* and NASM strains [31]. We did not observe an eradication effect of AgNPs synthesized from lavender extract in any of the tested strains, even at the highest tested concentrations. This ineffectiveness against formed biofilms will most likely be caused by the architecture of the biofilm, which consists of a matrix of extracellular polymeric substances. A possibility to disrupt the biofilm layer could be the use of a combination of several substances or a multiple increase in concentration above the MIC values [55].

In addition to AgNPs, we also tested the effect of EO obtained from lavender, with the aim of comparing these two different substances with the same biological origin. We were interested in whether LEO would be equally effective against all tested bacterial strains and in what concentrations. Based on our previous experience and published work [22] testing various EOs, we chose a concentration range using decimal dilutions from 1 to 0.0001%, which by weight represented 9010 to 0.901 μg/mL (9.01–0.901 μg/μL). As expected, the biological effect of the EO was higher at higher concentrations than that of AgNPs. The antimicrobial activity of LEO was tested by Stamova et al. [56] on various bacterial genera, including *S. aureus*. From the published results, the inhibition of bacterial growth was monitored using a 5% concentration, which represented the MIC value, which is five times higher compared to our concentration. This can be explained by the different representation of individual compounds resulting from the geographical origin of the plants used. More than twice the concentration for inhibiting the growth of *S. aureus* (MIC = 2000 μg/mL or 2 μg/μL) compared to ours (MIC = 901 μg/mL or 0.901 μg/μL) was also used in their work by de Rapper et al. [57]. At the same time, they pointed out effective synergism with conventional antimicrobial agents, thus clearly confirming the possibility of their mutual potentiation. When monitoring the inhibition of bacterial growth by LEO, we did not notice any differences in the effects between the individual staphylococci tested.

When monitoring antibiofilm activity, we noted a significant effect of LEO on biofilm formation at a ten-fold lower concentration than for inhibition of bacterial growth, which was at the level of 90.1 μg/mL (0.0901 μg/μL) for both *S. aureus* and NASM species. Significant inhibition of staphylococcal biofilm formation was also noted by Neisari et al. [58]; however, it was at much higher concentrations (1250 μg/mL). A good ability to inhibit biofilm formation in staphylococci by EOs was also observed by Manzoor et al. [59], who, based on comparison, determined the highest biofilm activity in clove EO, followed by lavender and the lowest in basil EO.

Although the green synthesis of silver nanoparticles using *Lavandula angustifolia* has previously been reported, the present study extends existing knowledge by providing a comprehensive comparative evaluation of lavender-derived AgNPs and commercial lavender essential oil against multidrug-resistant and biofilm-forming staphylococci, including clinically relevant NASM isolates. In addition to antibacterial and antibiofilm activities, the present study also evaluated the biofilm-eradicating potential of both alternative antimicrobial approaches, providing a more comprehensive biological characterization of their antimicrobial properties. Furthermore, our findings support future studies focused on elucidating the mechanisms of action and investigating synergistic antimicrobial strategies involving AgNPs, lavender essential oil and conventional antimicrobial agents.

An important limitation of the present study is that the long-term stability of the synthesized AgNPs was evaluated under storage conditions using UV–Vis spectroscopy, whereas the biological experiments were performed in complex microbiological media. It is well known that nanoparticle behavior may change considerably in biological environments due to aggregation, partial dissolution, or interactions with proteins and other medium components, which may influence their physicochemical properties and biological activity [60]. Therefore, further studies should investigate the stability and behavior of lavender-derived AgNPs directly in biologically relevant media to better understand their performance under experimental and potential biomedical conditions.

## 5. Conclusions

In this study, the biological activities of biosynthesized AgNPs prepared using *Lavandula angustifolia* extract and commercially available lavender essential oil (LEO) were comparatively evaluated. The obtained results demonstrated that biosynthesized AgNPs exhibited stronger antibacterial and antibiofilm activities than LEO, requiring lower effective concentrations against both reference strains and clinically relevant multidrug-resistant NASM isolates. However, neither AgNPs nor LEO exhibited sufficient biofilm-eradicating activity under the applied experimental conditions. These findings indicate that both alternative antimicrobial approaches represent promising candidates for further investigation rather than immediate therapeutic application. The observed biological activities suggest that combining AgNPs with LEO or conventional antimicrobial agents may enhance antibacterial efficacy through complementary mechanisms of action, including bacterial membrane disruption and improved antibiotic penetration. Such synergistic approaches may contribute to overcoming antimicrobial resistance and should therefore be investigated in future studies. Although the present study demonstrated significant antibacterial and antibiofilm activities under in vitro conditions, the precise molecular mechanisms responsible for these biological effects remain to be elucidated. Future research should focus on membrane damage, oxidative stress induction, biofilm-related gene expression, and the interaction of AgNPs with bacterial cells. Furthermore, comprehensive cytocompatibility studies and in vivo investigations are required to evaluate the efficacy, safety, pharmacokinetics and therapeutic potential of these materials before considering any biomedical or clinical application.

## Figures and Tables

**Table 1:** Chemical composition of LEO according to the manufacturer’s data sheet.

Chemical Composition	Specification	Result
Linalool	20–50%	43 ± 2%
Linalyl acetate	–	39 ± 2%

**Table 2:** Overall results of the biological activity of AgNPs and LEO against resistant and biofilm-forming *Staphylococcus* spp.

Bacterial Strain	Antibacterial Activity (AgNPs/LEO) *	Antibiofilm Activity (AgNPs/LEO) *	Biofilm-Eradication Effect (AgNPs/LEO) *
*S. aureus* CCM 4750	0.05/0.901	0.05/0.0901	-/-
*S. aureus* CCM 4223	0.05/0.901	0.05/0.0901	-/-
*M. sciuri*	0.025/0.901	0.025/0.0901	-/-
*S. pseudintermedius*	0.025/0.901	0.025/0.0901	-/-

Values represent the lowest tested concentrations showing statistically significant activity compared to the positive control (Dunnett’s test). * Values are expressed in µg/µL.

## Data Availability

The original contributions presented in this study are included in the article. Further inquiries can be directed to the corresponding author.

## Supplementary Materials

The following supporting information can be downloaded at: https://www.mdpi.com/article/10.3390/pharmaceutics18080930/s1, Supplementary Material Statement [35,36,61,62,63,64,65,66]. Figure S1: FTIR spectrum of the aqueous extract of *Lavandula angustifolia*; Figure S2: HPLC-DAD chromatogram of the *Lavandula angustifolia* extract recorded at 200 nm together with UV spectra of the identified compounds; Figure S3: Particle size distribution determined by DLS (a) and zeta potential (b) of silver nanoparticles, DLS measure (c) zeta potential measure (d); Figure S4: Size distribution of silver nanoparticles synthesized using *Lavandula angustifolia* extract; Figure S5: UV–Vis spectra of AgNPs colloids synthesized using *Lavandula angustifolia* extract monitored over 21 days. Table S1: Statistical analysis of antibacterial activity of AgNP; Table S2: Statistical analysis of antibiofilm activity of AgNP; Table S3: Statistical analysis of biofilm eradication effect of AgNP; Table S4: Statistical analysis of antibacterial activity of LEO; Table S5: Statistical analysis of antibiofilm activity of LEO; Table S6: Statistical analysis of biofilm eradication effect of LEO; Table S7: Physicochemical properties of AgNPs at 25 °C.

## Abbreviations

The following abbreviations are used in this manuscript:

AgNO_3_	silver nitrate
AgNPs	silver nanoparticles
AMR	antimicrobial resistance
ANOVA	analysis of variance
CLSI	Clinical and Laboratory Standards Institute
EDX	energy-dispersive X-ray spectroscopy
EOs	essential oils
EUCAST	European Committee on Antimicrobial Susceptibility Testing
LEO	lavender essential oil
mBHI	modified brain heart infusion broth
MDR	multidrug resistance
MIC	minimum inhibitory concentration
MRSA	methicillin-resistant *Staphylococcus aureus*
NaCl	sodium chloride
NASM	non-*aureus* staphylococci and mammaliicocci
NPs	nanoparticles
SD	standard deviation
SEM	scanning electron microscopy
SPR	surface plasmon resonance
TEM	transmission electron microscopy
WHO	World Health Organization
