# Supplementary material for: Comparison of the Effect of Silver Nanoparticles Biosynthesized with Lavandula angustifolia Extract and Lavender Essential Oil Against Multidrug-Resistant and Biofilm-Forming Staphylococcus Species
Source: Pharmaceutics. 2026 Jul 29;18(8):930. doi: 10.3390/pharmaceutics18080930 (PMC13516407; doi:10.3390/pharmaceutics18080930)
Supplement: Supplementary file 1 [file pharmaceutics-18-00930-s001.zip › pharmaceutics-4453909-supplementary.pdf]

Supplementary Materia Statement

This Supplementary Material contains additional characterization data of the *Lavandula angustifolia* extract used for the green synthesis of silver nanoparticles, including FTIR, ICP-OES and HPLC-DAD analyses.

FTIR Analysis

FTIR spectra were acquired using a Bruker Tensor 27 spectrometer (Bruker Optics, Germany) equipped with a DTGS detector. Samples were prepared by the KBr pellet method and measured in the 4000–400 cm^{-1} range with a resolution of 4 cm^{-1} using 64 scans per sample.

ICP-OES Analysis

The concentration of silver in the prepared AgNPs colloid was determined using inductively coupled plasma optical emission spectroscopy (ICP-OES). Measurements were performed using an ICP_PRO X Duo spectrometer (S/N ICAPPRO10652). Prior to analysis, the colloidal samples were appropriately diluted with deionized water. Silver concentrations were determined by external calibration using Ag standard solutions and expressed as ppm.

HPLC-DAD Analysis

The phytochemical profile of the *Lavandula angustifolia* extract was evaluated using gradient reversed-phase HPLC-DAD analysis. The samples were analysed using an Agilent 1260 Infinity Quaternary LC system equipped with a 1260 Infinity DAD detector (Agilent Technologies, USA). Separation was performed on an Arion® Polar C18 column (250 × 4.6 mm, 5 µm) at a flow rate of 1.0 mL min^{-1}. Compounds were monitored at 200 nm.

Analyses

To support the characterization of the plant extract used for the green synthesis of silver nanoparticles, FTIR, ICP-OES, and HPLC-DAD analyses were performed.

The FTIR spectrum of the *Lavandula angustifolia* extract (Figure S1) confirmed the presence of functional groups characteristic of phenolic compounds, flavonoids, organic acids, and other secondary metabolites, which are known to participate in the reduction of Ag^{+} ions and stabilization of the synthesized nanoparticles [61–63].

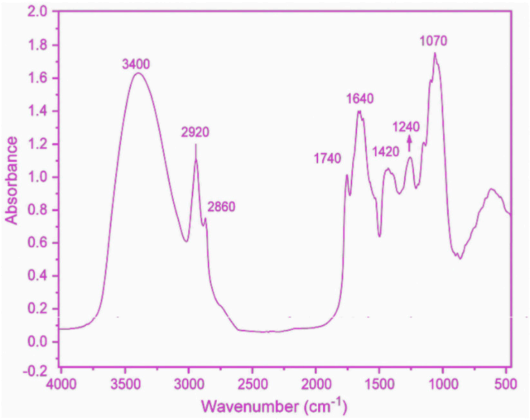

Figure S1. FTIR spectrum of the aqueous extract of *Lavandula angustifolia*.

Observed absorption bands at approximately 3400 cm^{-1} were assigned to the stretching vibrations of hydroxyl groups (O–H), whereas bands in the 2920–2958 cm^{-1} region corresponded to C–H stretching

vibrations of aliphatic compounds. Absorption bands in the 1700–1600 cm^{-1} region were associated with carbonyl groups (C=O), aromatic structures, and conjugated C=C bonds. Bands in the 1400–1000 cm^{-1} range were attributed to C–O and C–O–C stretching vibrations characteristic of polyphenols, flavonoids, and carbohydrates [62,64]. These functional groups likely participated in the reduction of Ag^{+} ions and the stabilization of the synthesized nanoparticles [35, 36, 65].

The successful synthesis of silver nanoparticles was further confirmed by ICP-OES analysis, which was used to determine the silver content in the prepared colloidal solution. ICP-OES analysis revealed a silver concentration of **469 ppm** in the biosynthesized colloidal suspension, confirming the efficient reduction of Ag^{+} ions by phytochemicals present in the lavender extract [36, 65].

Semi-quantitative HPLC-DAD analysis of the *Lavandula angustifolia* extract (Figure S2) revealed a complex profile of phenolic compounds and flavonoids. The predominant constituent was rosmarinic acid, accounting for approximately 18% of the relative peak area. Significant amounts of caffeic acid derivatives (~11%), 6-hydroxyluteolin-7-O-glycoside (~9%), and luteolin-7-O-glycosides (~7% each) were also detected. Minor constituents included vanillic acid, catechins, and rosmarinic acid derivatives. Peak assignment was performed by comparing retention times and UV spectra with literature data reported for lavender extracts [61,63, 66].

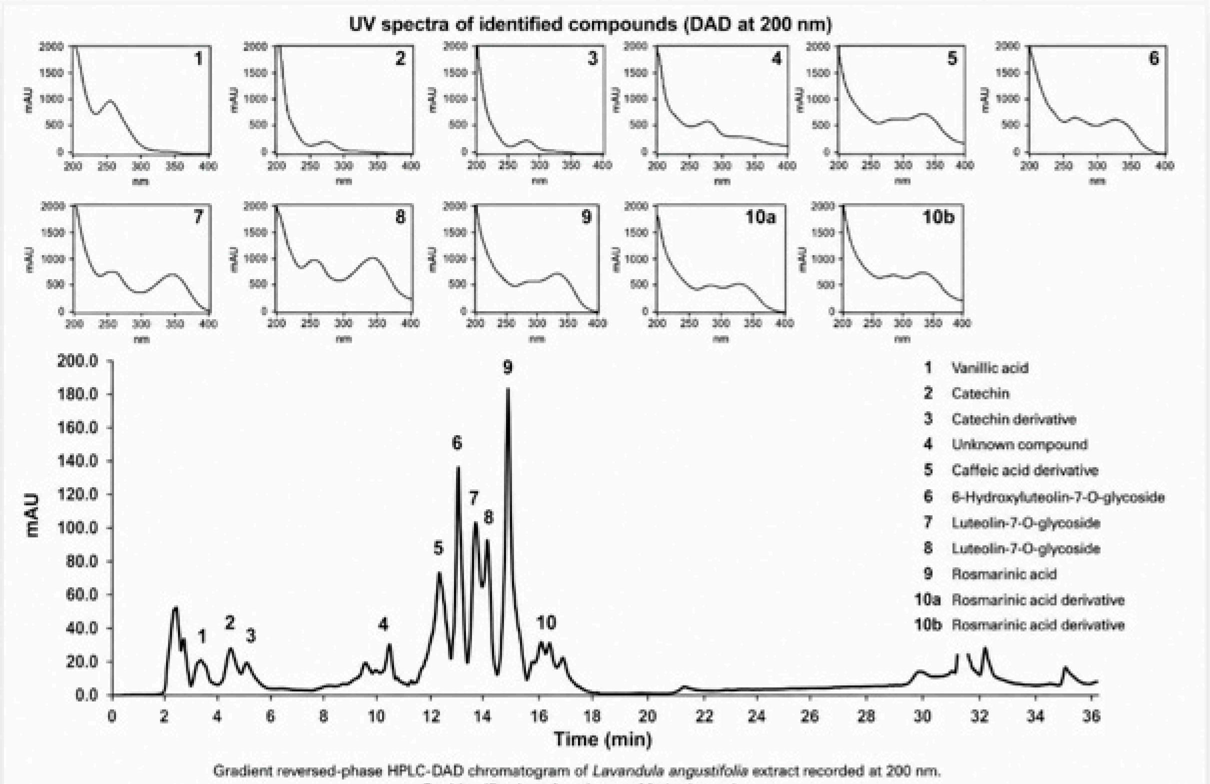

Gradient reversed-phase HPLC-DAD chromatogram of *Lavandula angustifolia* extract recorded at 200 nm.
For identification of peaks, see Methods section.

Figure S2. HPLC-DAD chromatogram of the *Lavandula angustifolia* extract recorded at 200 nm together with UV spectra of the identified compounds.

The identified composition indicates a high content of polyphenolic compounds with pronounced antioxidant properties [61,66]. Rosmarinic acid, caffeic acid derivatives, and flavonoids are known for their ability to donate electrons, thereby contributing to the reduction of Ag^{+} ions to metallic Ag^{0} during the green synthesis of nanoparticles [35, 36]. Furthermore, these biomolecules can adsorb onto the nanoparticle surface and act as natural stabilizing agents, thereby preventing nanoparticle aggregation [35].

The results of the HPLC-DAD analysis are in good agreement with the FTIR characterization of the extract, in which functional groups typical of phenolic acids and flavonoids were identified [1–3]. The predominance of rosmarinic acid suggests that this compound, together with other polyphenols, may play a crucial role in the reduction of Ag^{+} ions and the subsequent stabilization of biosynthesized silver nanoparticles.

Supplementary Tables S1–S6. Statistical analysis of the antibacterial, antibiofilm and biofilm eradication activities of AgNP and LEO

The supplementary tables summarize the statistical analyses corresponding to the data presented in Figures 5 – 10. Values are expressed as mean ± SD (n = 3) and statistical significance was evaluated using one-way ANOVA followed by Dunnett's test. Adjusted p-values represent comparisons between each tested concentration and the corresponding positive control.

Table S1. Statistical analysis of antibacterial activity of AgNP

Antibacterial activity of AgNP			
**Concentration of AgNP**	**Mean ± SD**	**One-way ANOVA (F; p-value)**	**Dunnett´s test (adjusted p value)**
***S. aureus* CCM 4750** (positive control)	2.386 ± 0.010	F (5, 12) = 2692, p < 0.0001	-
200 µg/mL	1.001 ± 0.017		p < 0.0001
150 µg/mL	1.301 ± 0.020		p < 0.0001
100 µg/mL	1.847 ± 0.027		p < 0.0001
50 µg/mL	2.083 ± 0.021		p < 0.0001
25 µg/mL	2.385 ± 0.015		p > 0.9999
***S. aureus* CCM 4223** (positive control)	2.219 ± 0.004	F (5, 12) = 1355, p < 0.0001	-
200 µg/mL	0.930 ± 0.017		p < 0.0001
150 µg/mL	1.085 ± 0.020		p < 0.0001
100 µg/mL	1.740 ± 0.027		p < 0.0001
50 µg/mL	2.083 ± 0.021		p = 0.0002
25 µg/mL	2.218 ± 0.015		p > 0.9999
S. sciuri (positive control)	2.458 ± 0.027	F (5, 12) = 1168, p < 0.0001	-
200 µg/mL	0.869 ± 0.044		p < 0.0001
150 µg/mL	0.915 ± 0.050		p < 0.0001
100 µg/mL	1.226 ± 0.040		p < 0.0001
50 µg/mL	1.958 ± 0.023		p < 0.0001
25 µg/mL	2.218 ± 0.008		p < 0.0001
S. pseudintermedius (positive control)	2.232 ± 0.005	F (5, 12) = 2341, p < 0.0001	-
200 µg/mL	0.601 ± 0.034		p < 0.0001
150 µg/mL	0.950 ± 0.027		p < 0.0001
100 µg/mL	1.469 ± 0.036		p < 0.0001
50 µg/mL	1.986 ± 0.015		p < 0.0001
25 µg/mL	2.107 ± 0.009		p = 0.0002

Table S2. Statistical analysis of antibiofilm activity of AgNP

Antibiofilm activity of AgNP			
**Concentration of AgNP**	**Mean ± SD**	**One-way ANOVA (F; p-value)**	**Dunnett´s test (adjusted p value)**
***S. aureus* CCM 4750** (positive control)	2.450 ± 0.051	F (5, 12) = 2464, p < 0.0001	-
200 µg/mL	0.643 ± 0.017		p < 0.0001
150 µg/mL	0.708 ± 0.020		p < 0.0001
100 µg/mL	0.916 ± 0.027		p < 0.0001
50 µg/mL	1.307 ± 0.021		p < 0.0001
25 µg/mL	2.414 ± 0.015		p = 0.4549
***S. aureus* CCM 4223** (positive control)	2.249 ± 0.071	F (5, 12) = 1245, p < 0.0001	-
200 µg/mL	0.558 ± 0.035		p < 0.0001
150 µg/mL	0.722 ± 0.003		p < 0.0001
100 µg/mL	0.849 ± 0.023		p < 0.0001
50 µg/mL	1.083 ± 0.026		p = 0.0002
25 µg/mL	2.250 ± 0.010		p = 0.9998
S. sciuri (positive control)	2.647 ± 0.036	F (5, 12) = 2734, p < 0.0001	-
200 µg/mL	0.525 ± 0.027		p < 0.0001
150 µg/mL	0.892 ± 0.039		p < 0.0001
100 µg/mL	0.983 ± 0.029		p < 0.0001
50 µg/mL	1.569 ± 0.010		p < 0.0001
25 µg/mL	2.519 ± 0.026		p = 0.0008
S. pseudintermedius (positive control)	3.361 ± 0.037	F (5, 12) = 6462, p < 0.0001	-
200 µg/mL	0.457 ± 0.009		p < 0.0001
150 µg/mL	0.628 ± 0.029		p < 0.0001
100 µg/mL	0.651 ± 0.013		p < 0.0001
50 µg/mL	0.757 ± 0.035		p < 0.0001
25 µg/mL	2.950 ± 0.034		p < 0.0001

Table S3. Statistical analysis of biofilm eradication effect of AgNP

Biofilm eradication effect of AgNP			
**Concentration of AgNP**	**Mean ± SD**	**One-way ANOVA (F; p-value)**	**Dunnett´s test (adjusted p value)**
***S. aureus* CCM 4750** (positive control)	2.514 ± 0.031	F (5,12) = 1.251, p = 0.3456	-
200 µg/mL	2.484 ± 0.010		p = 0.3379
150 µg/mL	2.485 ± 0.015		p = 0.3859
100 µg/mL	2.493 ± 0.009		p = 0.6530
50 µg/mL	2.508 ± 0.036		p = 0.9960
25 µg/mL	2.513 ± 0.007		p > 0.9999
***S. aureus* CCM 4223** (positive control)	2.852 ± 0.019	F (5,12) = 0.4275, p = 0.8210	-

200 µg/mL	2.833 ± 0.047		p = 0.7604
150 µg/mL	2.836 ± 0.009		p = 0.8358
100 µg/mL	2.842 ± 0.012		p = 0.9673
50 µg/mL	2.851 ± 0.009		p > 0.9999
25 µg/mL	2.851 ± 0.013		p > 0.9999
S. sciuri (positive control)	2.325 ± 0.034	F (5,12) = 0.3293, p = 0.8857	-
200 µg/mL	2.309 ± 0.016		p = 0.7730
150 µg/mL	2.314 ± 0.013		p = 0.9264
100 µg/mL	2.323 ± 0.016		p = 0.9997
50 µg/mL	2.324 ± 0.018		p = 0.9998
25 µg/mL	2.324 ± 0.018		p = 0.9999
S. pseudintermedius (positive control)	4.008 ± 0.041	F (5,12) = 1.829, p = 0.1816	-
200 µg/mL	3.957 ± 0.014		p = 0.0794
150 µg/mL	3.962 ± 0.018		p = 0.1202
100 µg/mL	3.968 ± 0.029		p = 0.2003
50 µg/mL	3.978 ± 0.012		p = 0.4185
25 µg/mL	3.983 ± 0.010		p = 0.5984

Table S4. Statistical analysis of antibacterial activity of LEO

Antibacterial activity of LEO			
**Concentration of LEO**	**Mean ± SD**	**One-way ANOVA (F; p-value)**	**Dunnett´s test (adjusted p value)**
***S. aureus* CCM 4750** (positive control)	0.881 ± 0.031	F (5, 12) = 575.9, p < 0.0001	-
1 %	0.343 ± 0.009		p < 0.0001
0.1 %	0.479 ± 0.016		p < 0.0001
0.01 %	0.874 ± 0.011		p = 0.9861
0.001 %	0.876 ± 0.012		p = 0.9949
0.0001 %	0.880 ± 0.019		p > 0.9999
***S. aureus* CCM 4223** (positive control)	0.853 ± 0.025	F (5, 12) = 349.2, p < 0.0001	-
1 %	0.526 ± 0.004		p < 0.0001
0.1 %	0.574 ± 0.011		p < 0.0001
0.01 %	0.844 ± 0.014		p = 0.8750
0.001 %	0.845 ± 0.010		p = 0.8896
0.0001 %	0.851 ± 0.005		p = 0.9997
S. sciuri (positive control)	0.418 ± 0.013	F (5, 12) = 37.64, p < 0.0001	-
1 %	0.322 ± 0.003		p < 0.0001
0.1 %	0.337 ± 0.022		p < 0.0001
0.01 %	0.417 ± 0.015		p > 0.9999
0.001 %	0.418 ± 0.009		p > 0.9999
0.0001 %	0.418 ± 0.006		p > 0.9999
S. pseudintermedius (positive control)	0.561 ± 0.006	F (5, 12) = 1402, p < 0.0001	-

1 %	0.185 ± 0.008		p < 0.0001
0.1 %	0.226 ± 0.010		p < 0.0001
0.01 %	0.552 ± 0.009		p = 0.5803
0.001 %	0.560 ± 0.009		p = 0.9999
0.0001 %	0.561 ± 0.009		p > 0.9999

Table S5. Statistical analysis of antibiofilm activity of LEO

Antibiofilm activity of LEO			
**Concentration of LEO**	**Mean ± SD**	**One-way ANOVA (F; p-value)**	**Dunnett´s test (adjusted p value)**
***S. aureus* CCM 4750** (positive control)	2.752 ± 0.017	F (5, 12) = 4568, p < 0.0001	-
1 %	0.543 ± 0.037		p < 0.0001
0.1 %	0.834 ± 0.029		p < 0.0001
0.01 %	0.844 ± 0.020		p < 0.0001
0.001 %	2.748 ± 0.039		p = 0.9997
0.0001 %	2.750 ± 0.020		p = 0.9999
***S. aureus* CCM 4223** (positive control)	2.752 ± 0.025	F (5, 12) = 3701, p < 0.0001	-
1 %	0.564± 0.008		p < 0.0001
0.1 %	0.634 ± 0.029		p < 0.0001
0.01 %	0.753 ± 0.034		p < 0.0001
0.001 %	2.257 ± 0.013		p = 0.4484
0.0001 %	2.258 ± 0.031		p = 0.4960
S. sciuri (positive control)	1.501 ± 0.016	F (5, 12) = 1663, p < 0.0001	-
1 %	0.438 ± 0.012		p < 0.0001
0.1 %	0.659 ± 0.033		p < 0.0001
0.01 %	0.861 ± 0.024		p < 0.0001
0.001 %	1.480 ± 0.019		p = 0.6040
0.0001 %	1.493 ± 0.010		p = 0.9764
S. pseudintermedius (positive control)	2.825 ± 0.034	F (5, 12) = 4500, p < 0.0001	-
1 %	0.682 ± 0.027		p < 0.0001
0.1 %	0.841 ± 0.033		p < 0.0001
0.01 %	2.096 ± 0.027		p < 0.0001
0.001 %	2.813 ± 0.017		p = 0.9678
0.0001 %	2.813 ± 0.011		p = 0.9713

Table S6. Statistical analysis of biofilm eradication effect of LEO

Biofilm eradication effect of LEO			
**Concentration of LEO**	**Mean ± SD**	**One-way ANOVA (F; p-value)**	**Dunnett´s test (adjusted p value)**
***S. aureus* CCM 4750** (positive control)	1.986 ± 0.030	F (5, 12) = 2.379, p = 0.1015	-

1 %	1.925 ± 0.011		p = 0.0565
0.1 %	1.932 ± 0.011		p = 0.0993
0.01 %	1.954 ± 0.022		p = 0.4807
0.001 %	1.965 ± 0.018		p = 0.8052
0.0001 %	1.971 ± 0.047		p = 0.9328
***S. aureus* CCM 4223** (positive control)	1.636 ± 0.036	F (5, 12) = 0.7330, p = 0.6125	-
1 %	1.605 ± 0.011		p = 0.4453
0.1 %	1.610 ± 0.022		p = 0.6069
0.01 %	1.618 ± 0.011		p = 0.8506
0.001 %	1.623 ± 0.017		p = 0.9528
0.0001 %	1.633 ± 0.038		p = 0.9998
S. sciuri (positive control)	1.931 ± 0.010	F (5, 12) = 0.3779, p = 0.8544	-
1 %	1.912 ± 0.047		p = 0.7110
0.1 %	1.913 ± 0.008		p = 0.7485
0.01 %	1.916 ± 0.004		p = 0.8307
0.001 %	1.918 ± 0.012		p = 0.9083
0.0001 %	1.925 ± 0.007		p = 0.9965
S. pseudintermedius (positive control)	3.065 ± 0.024	F (5, 12) = 1.600, p = 0.2336	-
1 %	3.025 ± 0.013		p = 0.2352
0.1 %	3.027 ± 0.028		p = 0.2598
0.01 %	3.030 ± 0.032		p = 0.3383
0.001 %	3.053 ± 0.022		p = 0.9611
0.0001 %	3.060 ± 0.025		p = 0.9988

To address this comment, the revised manuscript has been expanded to include DLS, zeta potential, and PDI measurements (**Figure S3** and **Table S7**).

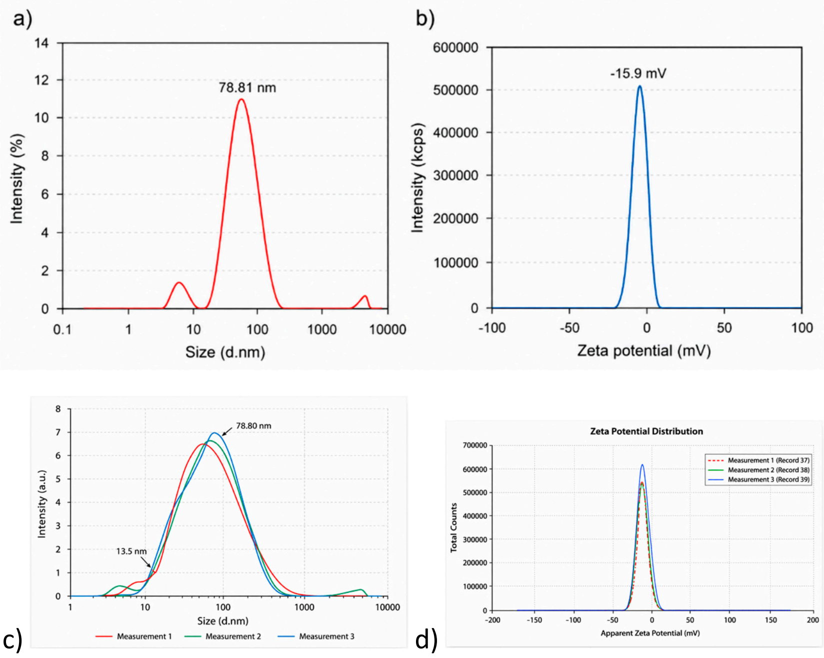

Figure S3. Particle size distribution determined by DLS (a) and zeta potential (b) of silver nanoparticles, DLS measure (c) zeta potential measure (d)

The biosynthesized AgNPs exhibited a hydrodynamic diameter of 76.89 nm, a zeta potential of −15.90 mV, and a PDI of 0.331 (Table S7), indicating a moderately polydisperse and sufficiently stable colloidal system suitable for biological applications.

Table S7. Physicochemical properties of AgNPs at 25 °C

TEMPERATURE (°C)	CONDUCTIVITY (MS/CM)	ZETA POTENTIAL (MV)	AVERAGE PARTICLE SIZE (NM)	POLYDISPERSITY INDEX (PDI)
**25**	0.751	−15.90	76.89	0.331

The particle size distribution obtained from DLS is consistent with the TEM analysis. Difference between DLS and TEM results is attributed to the hydrodynamic diameter measured by DLS, which includes the solvation layer and surface-bound phytochemicals.Particle size evaluation from TEM micrographs was performed using ImageJ software based on 12 independent TEM images, in which approximately 950 individual nanoparticles were analyzed. The resulting histogram confirms a relatively narrow size distribution with an average particle diameter of approximately 15–17 nm.

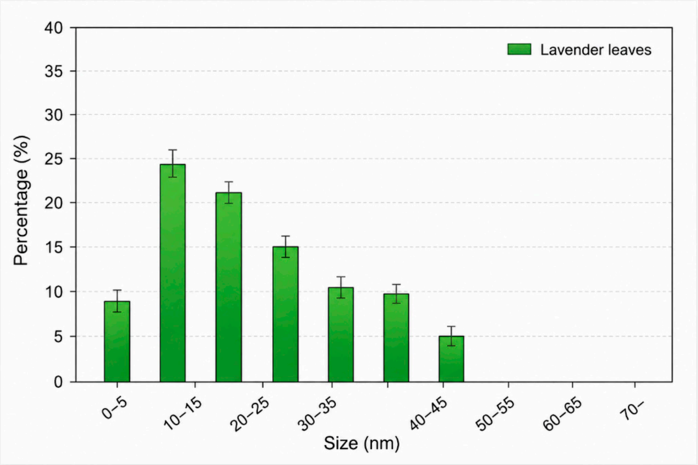

Figure S4. Size distribution of silver nanoparticles synthesized using *Lavandula angustifolia* extract.

Furthermore, the colloidal stability was independently verified by UV–Vis spectroscopy over a period of 21 days, Figure S3. No significant shift in the localized surface plasmon resonance (LSPR) absorption maximum was observed during storage, indicating the absence of significant nanoparticle aggregation.

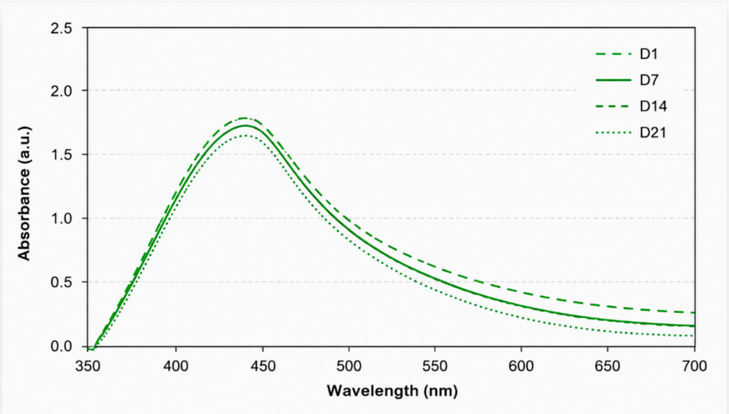

Figure S5. UV–Vis spectra of AgNPs colloids synthesized using *Lavandula angustifolia* extract monitored over 21 days.

These findings demonstrate that phytochemicals present in the *Lavandula angustifolia* extract act not only as reducing agents but also as effective natural stabilizing agents, ensuring sufficient colloidal stability throughout the antibacterial and antibiofilm assays.

Accordingly, the manuscript has been revised by incorporating DLS, zeta potential, PDI, particle size distribution analysis, and long-term UV–Vis stability data.
